# Language-driven anticipatory eye movements in virtual reality

**DOI:** 10.3758/s13428-017-0929-z

**Published:** 2017-08-08

**Authors:** Nicole Eichert, David Peeters, Peter Hagoort

**Affiliations:** 1 Max Planck Institute for Psycholinguistics, Nijmegen, The Netherlands; 2 University of Oxford, Oxford, UK; 3 Donders Institute for Brain, Cognition, and Behavior, Radboud University, Nijmegen, The Netherlands

**Keywords:** Virtual Reality, Prediction, Language Comprehension, Eyetracking, Visual World

## Abstract

Predictive language processing is often studied by measuring eye movements as participants look at objects on a computer screen while they listen to spoken sentences. This variant of the visual-world paradigm has revealed that information encountered by a listener at a spoken verb can give rise to anticipatory eye movements to a target object, which is taken to indicate that people predict upcoming words. The ecological validity of such findings remains questionable, however, because these computer experiments used two-dimensional stimuli that were mere abstractions of real-world objects. Here we present a visual-world paradigm study in a three-dimensional (3-D) immersive virtual reality environment. Despite significant changes in the stimulus materials and the different mode of stimulus presentation, language-mediated anticipatory eye movements were still observed. These findings thus indicate that people do predict upcoming words during language comprehension in a more naturalistic setting where natural depth cues are preserved. Moreover, the results confirm the feasibility of using eyetracking in rich and multimodal 3-D virtual environments.

**Electronic supplementary material:**

The online version of this article (doi:10.3758/s13428-017-0929-z) contains supplementary material, which is available to authorized users.

Prediction is a key feature of human cognition (Friston, 2010), and anticipatory behavior is steadily gaining the interest of researchers from different fields. The notion that we adjust certain actions on the basis of knowledge of upcoming events has been demonstrated in many experimental studies and has inspired theoretical and computational accounts of predictive information processing. Helmholtz (1860) had already incorporated probabilistic, knowledge-driven inference into his models of the human sensory systems. More elaborate theoretical and computational models of predictive processing evolved side by side with the experimental evidence, and Clark (2013, p. 1) even claimed brains to be “essentially prediction machines.” Psycholinguistics is one research area that is strongly concerned with different aspects of prediction (see Huettig, 2015). The predictive nature of language processing is a matter of ongoing debate, and recent studies have aimed to disentangle prediction from related concepts such as preactivation, anticipation, and integration (for a review, see Kuperberg & Jaeger, 2016).

A pivotal tool in the study of prediction in psycholinguistics has been eyetracking. In a seminal study performed over 40 years ago, Cooper (1974) investigated the role of eye movements during spoken language comprehension. Participants listened to short stories while looking at a visual display that depicted several objects. Their eye movements were recorded, and it was found that at remarkably short latencies, eye gaze was directed to those objects that were mentioned in the spoken sentences or that were associated with the content of the narrative. These findings led to the conclusion that eyetracking is a useful tool “for real-time investigation of perceptual and cognitive processes and, in particular, for the detailed study of speech perception, memory and language processing” (Cooper, 1974, p. 84). Further psycholinguistic studies elaborated on the paradigm introduced by Cooper, which was later termed the *visual-world paradigm* (VWP; see Huettig, Rommers, & Meyer, 2011; Tanenhaus, Spivey-Knowlton, Eberhard, & Sedivy, 1995).

In the typical screen-based “look-and-listen” variant of the VWP, participants are visually presented with line drawings or pictures of multiple objects on a computer screen. The auditory input in such studies is often a spoken word or a sentence that refers to the visual display in a certain manner defined by the experiment. An object that is directly mentioned in the spoken input is commonly referred to as the *target* object, whereas the other objects can be competitors or *distractor* objects. The underlying assumption of this variant of the VWP is that the auditory input is associated with a shift in attention that leads to an increased likelihood of fixating the target object relative to the other objects. Since the time to program a saccade can be reliably approximated as 200 ms (Matin, Shao, & Boff, 1993), eyetracking is a relatively precise method to study the timing of language comprehension in a visual context.

The use of the VWP has allowed researchers to draw important theoretical conclusions regarding the role of prediction in the online processing of spoken sentences. An influential eyetracking study in this domain was reported by Altmann and Kamide (1999), who presented participants with semirealistic scenes containing colored images of an agent (e.g., a boy), a target object (e.g., a cake), and several distractors (e.g., a ball, a toy car, and a toy train). While looking at the visual scenes on a computer screen, participants listened to simple spoken sentences that referred to the scene and the target object. Two experimental conditions were contrasted as a function of the relationship between the verb and the displayed objects. In the *restrictive* condition, the spoken sentence contained a verb that constrained the domain of subsequent reference so that only the target object could selectively be referred to by the verb (e.g., *The boy will* *eat* *the cake*, paired with a scene in which the cake is the only edible object). In the *unrestrictive* condition, the verb could relate to all presented objects (e.g., *The boy will* *move* *the cake*, paired with a scene in which all the depicted objects are moveable).

The resulting eye movement patterns showed that participants launched saccades to the target object significantly earlier in the restrictive than in the unrestrictive condition. Critically, this increased probability of looks to the target object was observed before the onset of the noun. These results therefore support the hypothesis that information encountered at the verb gives rise to anticipatory eye movements to possible visible referents, which indicated that listeners predict upcoming words. Altmann and Kamide (1999) indeed concluded that the brain can project an unrealized grammatical object based on verb-mediated knowledge in a given visual context (but see Yee & Sedivy, 2006). These findings are furthermore in line with work that has suggested that sentence processing happens in an incremental and piecewise manner (e.g., Tanenhaus et al., 1995) and stressed the fact that upcoming words are actively predicted by the processor (Altmann & Kamide, 1999).

In their experiments, researchers in psychology and cognitive neuroscience often make use of two-dimensional (2-D) line drawings and pictures that are mere abstractions of real-world objects (e.g., Huettig & McQueen, 2007; Snodgrass & Vanderwart, 1980). Limiting the complexity of stimuli in such a way increases experimental control over the variables of interest, but the generalizability of the results to everyday language processing remains debatable (Henderson & Ferreira, 2004). By using semirealistic visual scenes and colored clip-art objects, the study outlined above aimed to study sentence processing in relation to “real-world contexts” (Altmann & Kamide, 1999, p. 247). It is an open question, however, whether the anticipatory eye movements observed when testing participants that look at static, artificial images on a computer screen generalize to everyday situations of sentence processing in typical, naturalistic contexts (Henderson & Ferreira, 2004). To address this issue of ecological validity, more recent studies have investigated anticipatory eye movements as a proxy of prediction in language processing by using complex, realistic photographs of rich visual scenes as stimulus materials. These studies conceptually replicated the original effect (e.g., Andersson, Ferreira, & Henderson, 2011; Coco, Keller, & Malcolm, 2016; Staub, Abbott, & Bogartz, 2012). Another study, however, suggested that the ecological validity of the VWP is possibly limited to situations that present a relatively small number of distractor objects (Sorensen & Bailey, 2007).

In the present study, we focused on a different element that increases the ecological validity of an experimental visual stimulus—namely, its stereoscopic three-dimensional (3-D) presentation in an immersive 3-D visual context. After all, most objects we encounter in everyday communicative situations have at least three spatial dimensions. We adapted the variant of the VWP developed by Altmann and Kamide (1999) to be compatible with a virtual reality (VR) environment. VR environments preserve the stereoscopic depth cues that are inherent in naturalistic vision but have been absent in typical screen-based variants of the visual world paradigm. The simultaneous exposure to visual stimuli and related auditory input in VR leads to a more immersive character of the represented scene than traditional studies in which participants simply looked at a small computer monitor.

At a technical level, VR environments make use of various media in order to expose participants to a computer generated simulation. Though possibly all sensory modalities can be included, the visual and auditory domains are most commonly subject to virtual simulation (e.g., Slater, 2014). Stereoscopic vision and the percept of a 3-D space including depth are elicited by displaying two horizontally displaced images to the left and the right eye. In the present study, we presented the visual input by using projection screens in a cave automatic virtual environment (CAVE). A CAVE system consists of several projection surfaces that form a cubic space surrounding the participant (Cruz-Neira, Sandin, & DeFanti, 1993). Participants wear active shutter glasses that create a stereoscopic 3-D image by rapidly alternating between displaying and blocking the image intended for the respective eye. The timing of the alternation is coupled to the refresh rate of the projection screens, so that both devices work synchronously. Due to the high alternation frequency a coherently fused image is perceived. The glasses are furthermore part of a tracking system that monitors the position and direction of the participant’s head, controlling the correct perspective of the visual display.

Despite its powerful potential of combining experimental control and ecological validity, the use of VR in psycholinguistics has remained virtually nonexistent. Initial psycholinguistic studies using VR confirm the validity of this novel method by indicating that people speak to virtual interlocutors the way they speak to human interlocutors, and that they process speech produced by virtual agents in a similar way to that produced by human speakers. A study on language production in dialogue, for instance, demonstrated that natural linguistic-priming effects occur when participants interact with a human-like virtual agent (Heyselaar, Hagoort, & Segaert, 2017). It has also been found that participants accommodate their speech rate (Staum Casasanto, Jasmin, & Casasanto, 2010) and pitch (Gijssels, Staum Casasanto, Jasmin, Hagoort, & Casasanto, 2016) to the speech rate and pitch of their virtual interlocutors. Recent EEG evidence has suggested that similar cognitive and neural mechanisms may underlie speaking and listening to virtual as well as to human interlocutors (Peeters & Dijkstra, 2017; Tromp, Peeters, Meyer, & Hagoort, in press). These initial findings indicate the feasibility of using VR as a method to test whether traditional experimental findings may generalize to more naturalistic settings.

## The present study

The purpose of the present study was twofold. First, we aimed to conceptually replicate the findings of Altmann and Kamide (1999) in an immersive 3-D VR environment. This meant specifically that we tested for verb-mediated anticipatory eye movements to a visually presented target referent in a CAVE environment. Second, in doing so, we tested whether it is methodologically feasible to combine VR and eyetracking in the study of online language processing in a multimodal 3-D environment.

The conceptual replication we tested for in the present study was necessary before follow-up studies could start making use of the unique affordances of immersive VR in the domain of predictive language processing. If, for instance, a first VR study on predictive language processing in a visual environment were to make use of the full communicative, interactive, and audiovisual potential offered by this novel method, thereby finding results not in line with the original Altmann and Kamide (1999) claims, it would be unclear whether such a discrepancy were due to the VR method leading to different behavior than traditional methods or to the increase in ecological validity that the VR method affords. If the present study were to conceptually replicate the original findings, future studies could build on these results by using manipulations that can only be implemented in VR. The potential of VR lies in its increased ecological validity, as compared to screen-based studies. Rather than being a passive observer of stimuli on a computer screen, participants in a virtual environment themselves become part of the depicted scene. Whereas an increase in ecological validity often results in a decrease in experimental control, immersive VR has the potential to combine the naturalness of everyday interaction with a degree of experimental control that is to be desired by the experimental psychologist or cognitive neuroscientist.

**Fig. 1:**
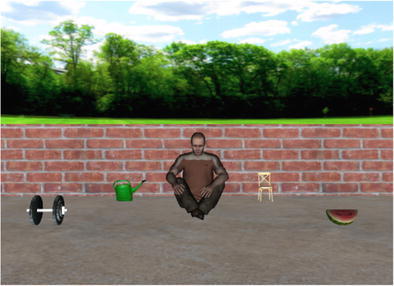
Example scene used in the experiment. Participants listened to the sentence *De man eet dadelijk de meloen* (“The man will soon eat the melon”) or *De man draagt dadelijk de meloen* (“The man will soon carry the melon”) while viewing the scene

Several changes were made to the original paradigm in order to avoid some confounding factors in the original study and to make the paradigm compatible with presentation in a 3-D virtual environment to Dutch participants. The most significant difference between the original study and our experiment was the mode of stimulus presentation. Altmann and Kamide (1999) had displayed the scenes on a relatively small computer screen (17 in.), and the stimuli were created using a 16-color palette. For the present study, we generated 3-D color objects rich in detail that were presented in an immersive virtual environment that features stereoscopic vision. Unlike in the original study, we aimed to keep the context information conveyed by the agent in the visual scenes minimal, because the identity of the agent can cause confounding predictive eye movements (Kamide, Altmann, & Haywood, 2003). A craftsman, for example, might be strongly associated with a machine-like object in the scene, regardless of the information extracted from the verb. Therefore we presented virtual agents that were of neutral appearance. Also in contrast to the original study, we made sure that the virtual agent was not looking at one of the objects, and we kept the number, animacy, and positions of objects per scene and relative to the background scenery constant. Furthermore, we controlled the verb materials for several linguistic features, including word length and frequency, to rule out that these parameters modulated the anticipatory effect. The present study was carried out in Dutch and with Dutch verbal materials (see Fig. 1 for an example). Dutch future tense places the (restrictive or nonrestrictive) target verb after the target noun, rendering the use of future tense impossible for investigating verb-based predictive processing in this version of the VWP. To assure that listeners would interpret each sentence as referring to an action that would take place in the future, the adverb *dadelijk* (“shortly, soon”) was included in each sentence (cf. Hintz, 2015). Finally, we doubled the number of trials in order to increase statistical power, and performed a finer-grained evaluation of eye movement patterns by implementing a logistic regression analysis that would overcome the problems associated with the use of traditional analyses of variance in analyzing eyetracking data.

We hypothesized that if the original findings of Altmann and Kamide (1999) generalized to situations of stereoscopic vision and immersed language processing, we should find anticipatory eye movements to the target object before noun onset for the restrictive condition only. The absence of such an effect would put in question whether the original findings can generalize to more naturalistic viewing conditions.

## Method

### Participants

Twenty-one native speakers of Dutch (19 female, two male; 19–27 years of age, mean age = 21.9) took part in the main experiment. The data of 30 participants was recorded, but nine were excluded due to the insufficient accuracy or quality of the eyetracking data. Participants were recruited via the online participant database of the Max Planck Institute for Psycholinguistics. They gave written informed consent prior to the experiment and were monetarily compensated for their participation. All participants had normal hearing and vision. The study was approved by the ethics board of the Social Sciences Faculty of Radboud University, Nijmegen, The Netherlands.

### Selection of stimulus materials

Thirty-two 3-D visual scenes were designed and were each paired with two spoken sentences. The scenes displayed a computer-generated virtual agent sitting in a backyard surrounded by four objects (see Fig. 1 for an example).

The virtual agent represented either a female or a male person. Both virtual agents were adapted from a stock avatar produced by WorldViz (2016) and appeared to be Caucasians in their mid-twenties (sportive06_f_highpoly and casual13_m_highpoly). The virtual agents were sitting crossed-legged on the virtual floor, and their facial expression was a modest smile. The gaze of the virtual agent was directed to the virtual floor between the agent and the participant, without showing a preference for looking at any of the objects. The virtual background environment showed a simple backyard scenery with brick walls and surrounding trees.

The experimental spoken sentences were in Dutch and had a simple subject–verb–adverb–object structure, such as *De man eet dadelijk de meloen* (“The man will soon eat the melon”). All sentences described an action that could apply to the presented visual display, and for each scene the two sentences differed only with respect to the verb. Depending on the presentation of a female or a male virtual agent, the subject of the sentence was changed accordingly (*de man* “the man” or *de vrouw* “the woman”).

To contrast two experimental conditions, we generated verb pairs that consisted of a *restrictive* and an *unrestrictive* verb. A restrictive verb imposed constraints on its arguments such that only one of the visually presented objects was a plausible argument. In the following, this object will be referred to as the *target object* of the scene. In the example illustrated in Fig. 1, the melon is the target object, because among the four visible objects, it is the only appropriate argument for the verb *to eat*. The unrestrictive verb in this scene, *to carry*, does not narrow down the domain of subsequent reference, because all four presented objects can function as plausible arguments.

Sixty-four verbs were selected from the Dutch Lexicon Project 2 database (DLP2; Brysbaert, Stevens, Mandera, & Keuleers, 2016), which contains several lexical measures for 30,000 Dutch lemmas, including length and frequency (see all measures listed in Appendix 1). The verbs were equally distributed over the restrictive and unrestrictive conditions, so that the linguistic features of the infinitive verb forms in the two conditions were comparable. We controlled for lexical characteristics obtained from the database and computed statistical comparisons in R (R Development Core Team, 2015). To obtain *p* values, we first assessed the assumptions for a Student’s *t* test by applying a Shapiro–Wilk test and an *F* test in order to check for normal distribution and equal variances. A two-tailed *t* test was performed if the measures met the statistical assumptions; otherwise, a Wilcoxon rank sum test was used. The measures for the mean and standard deviation (*SD*) or—for nonnormal parameters—the median and median absolute deviation are provided in Appendix 1, together with the corresponding *p* values. The verb pairs are listed in Appendix 2.

For each verb pair, we selected four names of objects that could function as grammatically correct postverbal arguments within the sentence (see Appx. 2). The four object names for a given scene were selected to be of the same grammatical gender, such that the Dutch definite article (*de* or *het*) would correspond to all objects within the scene. This ruled out the determiner interfering with the possible prediction formed on the basis of the verb. For each scene, only one of the four objects was a plausible argument for the restrictive verb. This object was the target of the scene, whereas the other three objects were distractors. The relation between the restrictive verb and the distractors either was not in accord with real-world experience or was highly unlikely. The unrestrictive verb did not impose any semantic restrictions on the argument in such a way that the target and distractors could equally likely be referred to as possible arguments of the verb. Consider, for example, the verb pair *eat/carry* and the objects melon, watering can, chair, and barbell. Since the melon is the only edible object among the four, it is considered the target object. All four objects, however, can be regarded as plausible arguments for the unrestrictive verb *to carry*.

The object names within one scene started with different phonemes in order to avoid phonological activation of the distractor objects. Allopenna, Magnuson, and Tanenhaus (1998) showed increased fixation probabilities for distractors that began with the same onset and vowel as the target object. Semantic relatedness is another confounding factor that has been shown to influence fixation behavior (Yee & Sedivy, 2006). For the given paradigm, however, it was impossible to completely avoid thematic associations between the four objects. Since all objects could serve as the argument for the unrestrictive verb, they shared at least one semantic feature with respect to their relation to the unrestrictive verb. For example, the verb *proeven* (“to taste”) is only plausibly related to food items, and objects for the verb *reinigen* (“to clean”) are commonly associated with objects in the household.

Semantic similarity has been shown to predict language-mediated eye movements in visual-world studies (Huettig, Quinlan, McDonald, & Altmann, 2006). To support our selection of object names, we measured the relatedness of verb–object pairs on the basis of semantic spaces. The semantic distance for the eight possible verb–object combinations within each scene were computed using the open-source web tool *snaut* (http://zipf.ugent.be/snaut-dutch; Mandera, Keuleers, & Brysbaert, 2017). The *snaut* tool computes a measure of semantic relatedness based on the count of co-occurrences of two lemmas in a large corpus. This principle is adapted from latent semantic analysis (Landauer & Dumais, 1997), which is a method for measuring semantic similarity of texts using corpus-based measures. The algorithm underlying *snaut* is a predictive neural network that makes use of a Continuous Bag of Words model (CBOW) architecture. Co-occurrences are obtained within a window of ten words that slides through the entire corpus. By adjusting weights, the network learns which words are related to each other. The relatedness of words is quantified by calculating the cosine distance of the two vector representations within a 200-dimensional space. The toolbox makes use of the Dutch SONAR-500 text corpus (Oostdijk, Reynaert, Hoste, & Schuurman, 2013) and a corpus of Dutch movie subtitles.

**Fig. 2:**
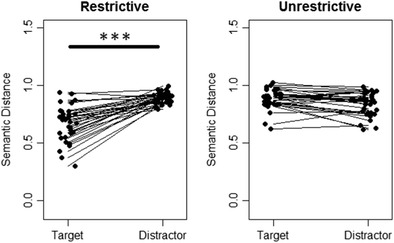
Semantic distances measured with the *snaut* tool. The data points for the target represent the semantic distance of single-word pairs (e.g., “restrictive verb + target”), and the data points for the distractors represent mean values of the three distractor word pairs in one scene. Pairwise comparison of the target and distractor pairs in the restrictive condition revealed a significant difference (paired Wilcoxon test, *n* = 64, *p* < .001)

We compared the values for the “restrictive verb + target” pairs to the mean values of the three “restrictive verb + distractor” pairs, pair-wise for each scene. The semantic distance of the word pairs in the restrictive condition differed significantly (paired Wilcoxon test, *n* = 64, *p* < .001). Comparing word pairs from the unrestrictive condition revealed no significant difference (paired Wilcoxon test, *n* = 64, *p > .*05). The semantic distances are illustrated in Fig. 2. The semantic relatedness results show that the restrictive verbs were semantically more closely related to the target than to the distractor objects. For unrestrictive verbs, the semantic distances in the two object categories did not differ. This pattern of semantic similarity was the essential characteristic defining the two experimental conditions. In the restrictive condition, only the target object was a plausible argument for the verb, implying a closer semantic relationship between verb and object. This result thus confirms that our selected combinations of verbs, targets, and distractors were suitable for the experimental questions we wanted to assess.

### Sentence recordings and annotation

The sentences were spoken by a female native speaker at a normal rate with neutral intonation. The recording was performed in a soundproof booth, sampled at 44.1 kHz (stereo, 16-bin sampling resolution), and stored digitally on computer. The audio file was chopped into individual audio files for each sentence using Praat (Boersma & Weenink, 2009), and all files were equalized for maximal amplitude. Sentences were annotated by placing digital markers at the onsets and offsets of critical words: verb onset, verb offset, determiner onset, and noun onset. The mean duration of the sentences was 3,065 ms (*SD* = 314), and the positions of the markers did not differ between the sentences of the restrictive and unrestrictive conditions (see Table 1).

**Table 1:** Comparison of the sentences in the two experimental conditions (restrictive and unrestrictive) for the critical on- and offset time points

	Verb Onset	Verb Offset	Determiner Onset	Noun Onset	Total Duration
Restrictive	728 (128)	1,299 (162)	2,103 (256)	2,244 (268)	3,048 (319)
Unrestrictive	730 (115)	1,315 (182)	2,132 (289)	2,263 (294)	3,082 (313)
*p* Value	.92	.62	.56	.71	.54

Mean values and standard deviations (in parentheses) are provided in milliseconds. All *p* values were obtained using two-tailed Student’s *t* tests.

### *3*-D virtual objects

A graphics designer created 3-D objects for the virtual environment using the 3-D computer graphics software Autodesk Maya (Autodesk Inc., 2016). The 128 objects were designed to represent a stereotypic instance of the objects that we had selected for the stimulus set. The texture that was added to the objects surface was either custom-made in the graphics software or taken from freely available pictures from the Internet.

Objects were presented as much as possible at their expected real-world size, but in certain cases they had to be scaled to account for the influence of object size on visual attention. Larger objects attract more visual attention than smaller objects, and are therefore more likely to be fixated by chance. We thus aimed to keep the sizes of the target and distractor objects comparable. We quantified object size by measuring the volume of a virtual bounding box, which is the regular cuboid including the entire object. The perceived size of an object changes depending on its position in the virtual scene, but the size of the bounding box is a constant value of the item. The volume of the target objects did not differ from the average volume of the three distractor objects (two-tailed, paired Student’s *t* test on logarithmic values, *n* = 32, *p* = *.*34).

**Fig. 3:**
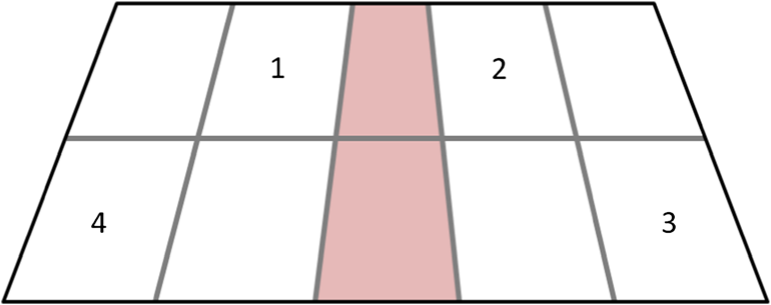
Hypothetical grid on the virtual ground used for the main experiment. The numbers indicate the positions of the four objects. The virtual agent was located in the middle of the screen and occupied two subspaces (red shading) where no objects were located. The grid was not visible during the experiment

The positions of the objects were determined on the basis of a hypothetical grid on the virtual ground, represented in Fig. 3. The virtual space in the computer software is described by means of a coordinate system wherein the *x*-axis represented the horizontal dimension, the *y*-axis the vertical dimension, and the *z*-axis the depth. The root of the coordinate system (0/0) was defined as the point of the middle line that was “closest” to the observer. The exact *x*- and *y*-positions of the four objects were (–1/3.8) for Object 1, (1/3.8) for Object 2, (1.5/2.2) for Object 3, and (–1.5/2.2) for Object 4. The space in front of and behind the virtual agent was never occupied by an object. Due to the 3-D perspective, items placed behind the agent would be at least partly hidden, and objects located in front of the agent would attract disproportionately high attention.

### Pretest: Identifiability of 3-D virtual objects

Twelve native speakers of Dutch (10 female, two male; 20–29 years of age, mean age = 24.1; they did not participate in the main experiment) took part in a naming pretest that was conducted to ensure that the 3-D objects were identifiable in VR. Thirty-five virtual scenes were presented in a virtual environment that was similar to that of the main experiment. Each scene thus displayed a virtual agent sitting in a backyard surrounded by four objects. In total, 140 objects were presented to the participants. The order of trials was randomized across participants, and the gender of the virtual agent was alternated across trials. We chose a stimulus display similar to that in the main experiment to assess the identifiability of the objects when they are presented in sets of four together with a virtual agent. Each object was randomly assigned to one of eight positions on the virtual ground (see Fig. 3).

The participants in the pretest were seated in a comfortable chair in the middle of the CAVE system, and a laptop for typing their answers was placed on their lap. During the pretest they wore the VR glasses, softly fastened using a strap on their head to ensure stability, and calibrated by a single calibration step for the head-tracking signal. They were instructed to type the four object names for each trial into the laptop. For recording their answers, we used a custom-made MATLAB script (The MathWorks Inc 2013), which prompted the participants to enter their answer for each scene one after the other. Typing in the answers was self-paced, and the participants indicated that they were ready for the next trial by raising their hand. During completion of the task, the participant was alone in the CAVE, but the experimenter was able to give further instructions, if necessary, via a microphone in the control room.

Answers were manually coded offline with regard to the correct identifiability of the object and the preferred object name. Objects that were incorrectly named by more than 25% of the participants (i.e., four out of the 12) were excluded from the stimulus set. If different synonymous names were given as answers, we selected the object name that was used by the majority of the participants. A set of 128 suitable object names was then selected for the main experiment so that the criteria described above were met (e.g., no overlap in the first phoneme for objects in the same scene). Eight objects with insufficient identifiability were selected to serve for the practice trials in the main experiment. The final set of object names is listed in Appendix 2, together with the corresponding verb pairs.

### Apparatus

#### The CAVE system

**Fig. 4:**
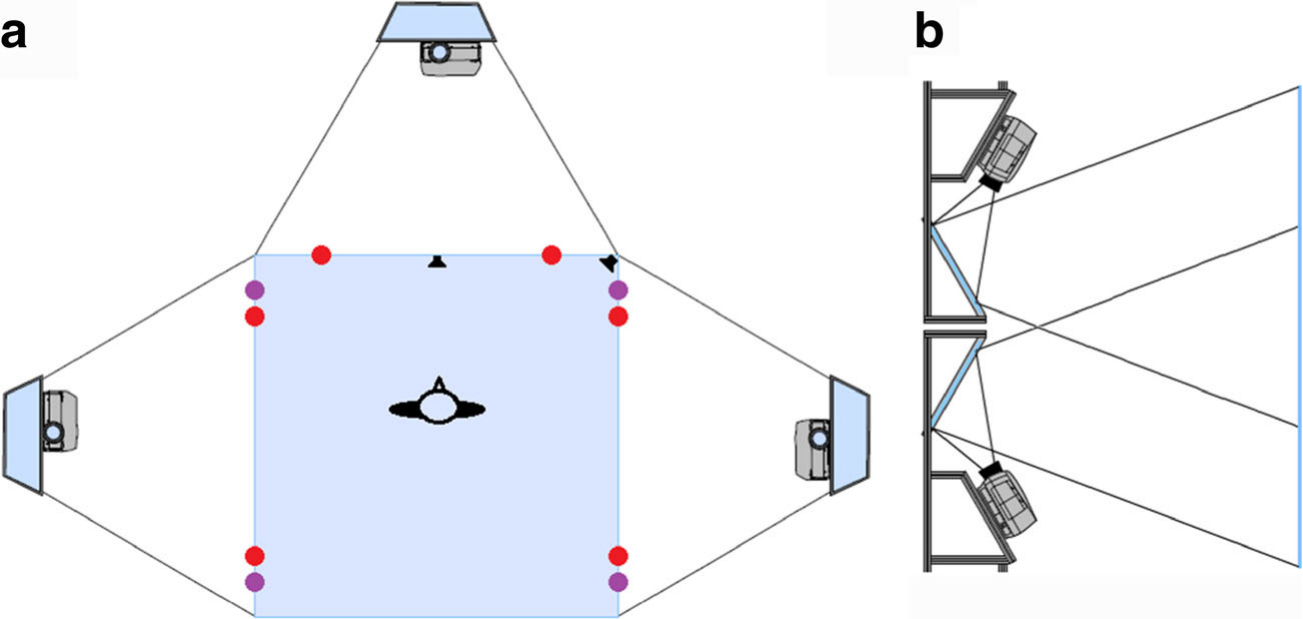
Schematic drawing of the CAVE system. (**a**) Top view indicating the configuration of the screens, the position of the participant, the infrared motion capture cameras, and the speakers. Red points represent cameras located at the upper edges of the screens, and purple points represent cameras at the bottom edges. The lower projectors are depicted for illustration purposes only. (**b**) Side view of one pair of projectors that illuminate the screen indirectly, via mirrors

The CAVE system consisted of three screens (255 × 330 cm, VISCON GmbH, Neukirchen-Vluyn, Germany) that were arranged at right angles as illustrated in the schematic drawing in Fig. 4. Two projectors (F50, Barco N.V., Kortrijk, Belgium) illuminated each screen indirectly through a mirror behind the screen. The two projectors showed two vertically displaced images that overlapped in the middle of the screen (see Fig. 4b). Thus, the complete display on each screen was only visible as the combined overlay of the two projections.

For optical tracking, infrared motion capture cameras (Bonita 10, Vicon Motion Systems Ltd, UK) and the Tracker 3 software (Vicon Motion Systems Ltd, UK) were used. The infrared cameras detected the positions of retroreflective markers by optical–passive motion capture. Six cameras were positioned at the upper edges of the CAVE screens, and four cameras were placed at the bottom edges. All cameras were oriented toward the middle of the CAVE system. The positions of the cameras are indicated in Fig. 4a.

Participants were sitting in a chair in the middle of the CAVE system so that the three screens covered their entire horizontal visual field. The eyes of the participant were approximately 180 cm away from the middle screen, so that 90° of the vertical visual field were covered by the display. The four objects that were presented in each virtual scene extended across approximately 80° of the horizontal visual field. The control room was located next to the experimental room containing the CAVE system. The experimenters could visually inspect the participant and the displays on the screens through a large window behind the participant.

The experiment was programmed and run using 3-D application software (Vizard, Floating Client 5.4, WorldViz LLC, Santa Barbara, CA), which makes use of the programming language Python. Spatial coordinates and distances in the VR environment are expressed as dimensionless numbers. The software translates the numbers one-to-one into virtual meters, but due to the adjusted object sizes, the numbers can be understood as relative rather than absolute measures.

Sound was presented through two speakers (Logitech, US) that were located at the bottom edges of the middle screen at the positions indicated in Fig. 4. The auditory signal was detected by a custom-made NESU-Box (Nijmegen Experiment Set Up, serial port), so that the on- and offset of the sentence were online recorded in the data stream.

#### Eyetracking

Eyetracking was performed using special glasses (SMI Eye-Tracking Glasses 2 Wireless, SensoMotoric Instruments GmbH, Teltow, Germany) that combine the recording of eye gaze with the 3-D presentation of VR. The recording interface used is based on a Samsung Galaxy Note 4 that is connected to the glasses by cable. The recorder communicates with the externally controlled tracking system via a wireless local area network (wifi), which enables live data streaming.

The glasses were equipped with a camera for binocular 60-Hz recordings and automatic parallax compensation. The shutter device and the recording interface were placed on a table behind the participants during recording. Gaze tracking accuracy was estimated by the manufacturer to be 0.5° over all distances. We found the latency of the eyetracking signal to be 200 ± 20 ms.

By combining eyetracking and optical head-tracking, we were able to identify the exact location of the eye gaze in three spatial dimensions, allowing participants to move their heads during the experiment. Optical head-tracking was accomplished by placing light reflectors on both sides of the glasses. Three spherical reflectors were connected on a plastic rack and two of such racks with a mirrored version of the given geometry were manually attached to both sides of the glasses using magnetic force. The reflectors work as passive markers that can be detected by the infrared tracking system in the CAVE. The tracking system was trained to the specific geometric structure of the three markers and detected the position of the glasses with an accuracy of 0.5 mm.

Calibration of the eyetracker required two separate steps: One for the position of the head within the optical tracking system and one for the position of the pupil monitored by the camera within the eyetracking glasses. For the calibration procedure we used a virtual test scenery. This environment resembled the inside of an Asian tea house and three colored spheres were displayed in front of the participants. The position of the tree spheres differed in all three spatial coordinates.

During the first calibration step, participants were asked to look at the three displayed spheres successively. The experimenter was present in the CAVE system and the calibration scene was also displayed on the recording interface. The experimenter selected the corresponding sphere that was fixated by the participant on the recording interface. The second calibration step was performed using Vizard software in the control room. The same test scenery and instructions were used and the experimenter could communicate with the participants via the microphone. The computer software computed a single dimensionless error measure of the eyetracker combining the deviance in all three coordinates. The computer-based calibration was repeated until a minimal error value (<5), and thus maximal accuracy, was reached.

The accuracy of the eyetracker could not be assessed quantitatively. An error message occurred during the initial calibration step if the eyetracker failed to detect the pupil with sufficient accuracy. In these cases, the participants were excluded from the experiment. Retrospective assessment of the tracking quality for each participant was performed using custom-made playback software. The software illustrated the movement of the recorded gaze position in a 3-D computer display together with the corresponding 3-D scenery. This display mode was used to visually inspect calibration quality and accuracy. Calibration quality was assessed by inspecting the deviation of the gaze position during the presentation of the fixation cross. Low tracking accuracy showed up as unstable and irregular movements of the gaze position. At this point, it is unclear whether the relatively high number of participants who had to be excluded was due to a difference in eyetracker quality between the present and previous studies or whether we simply happened to recruit a relatively high subset of participants with pupils that would have been hard to detect by any eyetracker.

#### Regions of interest

To determine target fixations, we defined individual 3-D regions of interest (ROIs) around each object in the virtual space. The *x* (width) and *y* (height) dimensions of the ROI were adopted from the frontal plane of the object’s individual bounding box, facing the participant. We adjusted the size of this plane to ensure a minimal size of the ROI. The minimal width was set to 0.8 and the minimal height to 0.5. For the presented layouts of objects, the adjusted *x* and *y* dimensions were sufficient to characterize the ROIs. Despite the 3-D view, the plane covered the whole object sufficiently to capture all fixations. The *z* dimension (depth) of the ROI was therefore set to a relatively small value of 0.1. An increased *z* value of the ROIs would not have been more informative about the gaze behavior, but it would have led to overlapping ROIs in some cases. The eyetracking software automatically detected when the eye gaze was directed at one of the ROIs and coded the information online in the data stream. Some previous studies have used the contours of the objects to define ROIs, but rectangles have been shown to produce qualitatively similar results (Altmann, 2011).

### Design and procedure

The participants in the main experiment were seated in a comfortable chair in the middle of the CAVE system and were familiarized with the upcoming procedure. They put on the VR glasses, which were softly fastened using a strap on their head to ensure stability. Prior to the start of the experiment, we performed the two calibration steps as described above. The calibration screen was furthermore used to test whether the stereoscopic display produced by the shutter glasses was working correctly.

Participants were asked to remain seated during the experiment and not to move the glasses. No specific instructions were given, besides to carefully listen to the sentences and to look at the display. Unlike typical eyetracking experiments, they were allowed to move their head. The experimental trials were preceded by two practice trials. Between trials, the empty virtual environment was presented without objects and virtual agent, but with a central fixation cross at the position of the agent’s head. The cross appeared for 1 s and participants were asked to fixate on the cross, whenever it appeared. The scene was presented for a preview time of 2 s before the audio file was played. The preview time ensured that participants had enough time to encode visual information and generate expectations (Huettig & McQueen, 2007) despite the unfamiliar setting in a VR environment.

Participants were presented with two experimental blocks of 32 trials each. The second block contained trials with the reversed condition (restrictive vs. unrestrictive verb), such that each participant was exposed to all of the possible 64 experimental trials. Between the two blocks, recalibration was performed using the Vizard software. The experimental condition for each trial was determined on the basis of a pseudo-randomization procedure. Four lists of trials were generated. Each participant was assigned to one of the four lists, such that the design was counterbalanced with respect to the experimental condition, gender of the virtual agent, and experimental block. The order of trials within each block was randomized while keeping the gender of the virtual agent alternating.

After the experiment, participants underwent a short debriefing interview to assess whether they had recognized the experimental manipulation. They were then informed about the actual aim of the study. Moreover, we informed them about the fact that we had recorded their eye movements, and all participants gave consent to use their eyetracking data for the purpose of the present study.

As we outlined above, the second block presented participants with the same visual scenes as in the first block, but with the verb form from the opposite experimental condition. The debriefing revealed that participants had noticed this, which led to an increase in fixations on the target object even before verb onset. Therefore, we restricted the main data analysis to Block 1. The analysis of Block 2 nevertheless showed the same critical effect preceding noun onset (see the supplementary materials).

### Statistical analyses

Although many visual-world studies have analyzed eyetracking data using *t* tests and analyses of variance (ANOVAs), the data usually violate the underlying statistical assumptions for such tests (Jaeger, 2008). The fundamental problem is that ANOVAs are designed to test the effect of a categorical variable on a continuous variable. Most visual-world studies, however, assess the effect of a continuous temporal phenomenon (e.g., spoken language) on a categorical variable (e.g., a fixated object). For the sake of ANOVAs, time is often transformed into a categorical variable by collapsing the data into a series of time windows (e.g., Hanna, Tanenhaus, & Trueswell, 2003) and aggregating over trials and subjects. Collapsing data into time windows can, however, obscure effects such as anticipatory eye movements and other dynamics. Furthermore, gaze behavior is usually coded as a binary variable (0 = “ROI not hit” and 1 = “ROI hit”) and then transformed into a continuous variable by calculating fixation proportions. The proportions and their confidence intervals are only defined on a range from 0 to 1. ANOVAs, however, assume unbound and homogeneous variances and might therefore produce spurious results (Jaeger, 2008).

Regression models have been shown to be a more appropriate framework for analyzing eyetracking experiments. They capture the temporal dynamics of the gaze behavior by treating time as a continuous variable. We transformed the dependent variables in the regression analysis using an empirical logit link function, which is the appropriate scale for assessing effects on a binary categorical dependent variable (Barr, 2008). The empirical logit is an approximation of the log odds transformation, which allows for a tolerance such that infinity is not returned when the argument is 0 or 1. Specifically, we performed a weighted linear regression over empirical logits (Barr, 2008).

Because we designed a within-subjects and within-item experiment, a multilevel logistic regression was performed. In a mixed-effect approach, nonindependence on the level of subjects and items was modeled by means of random effects. This approach makes it possible to control for their associated intraclass correlation, such as with random intercepts. The data for different trials and subjects do not need to be pooled together, as is traditionally done using ANOVAs. As a fixed effect in the main analysis, we modeled condition (restrictive vs. unrestrictive), time (as bin), and their interaction. Statistics were calculated using mixed-effects models from the lme4 package (Baayen, Davidson, & Bates, 2008) in the R environment. In the Results section, we report the parameter estimate (*Est*), standard error (*SE*), and *p* value for effects of interest. The variables condition and bin were contrast-coded to facilitate the interpretation of possible interactions and the directions of the effects. To avoid power reduction due to collinearity, we centered both variables by mean subtraction.

Data were acquired at a sampling frequency of 60 Hz, which means that approximately every 17 ms one sample was recorded. We corrected for the 200-ms latency shift caused by the eyetracking system by time-locking the data to 200 ms (12 samples) after sentence onset. As a variable of interest we defined the proportion of fixations on the target object. A fixation was defined as a look at the same ROI that lasted at least 100 ms—that is, six subsequent samples were coded as “hits” for the same ROI (see, e.g., Ettinger et al., 2003; Manor & Gordon, 2003; Sanchez, Vazquez, Gomez, & Joormann, 2014; Sekerina, Campanelli, & Van Dyke, 2016). This correction to the whole experimental dataset led to the exclusion of 2.8% of all samples in which a “hit” to a predefined ROI was detected. The fixation data were then aggregated into time bins of 50 ms (i.e., three samples) by participant, trial, and condition.

For the main analysis, we assessed the effect of the experimental condition (restrictive vs. unrestrictive) on the proportions of target fixations over a critical time window of 1.5 s that spanned from 200 ms (i.e., 12 samples) after verb onset until the average noun onset. The onset of the critical time window was based on previous evidence about saccadic planning (Matin et al., 1993); 200 ms after verb onset is the earliest point at which the linguistic stimulus can drive fixations to the target object. We further assessed the validity of this starting point by visually inspecting the grand means of the fully aggregated dataset.

Prior to the main analysis on the critical time window, we eliminated fixations that had been initiated before the onset of the verb and that extended into that time window, as had previous studies (e.g., Altmann & Kamide, 1999). This correction led to the elimination of 9.6% of all saccades. Moreover, we analyzed baseline effects to check whether any confounding effect was present in the baseline period before the experimental manipulation.

## Results

### Main analysis

For the main statistical analysis, we defined a critical time window in which we expected the experimental manipulation to have an effect on the proportions of target fixations. We chose the onset of the critical window as 200 ms after verb onset, assuming that it takes approximately 200 ms to plan and initiate a saccadic movement (Matin et al., 1993). As the offset of the critical time window, we chose the average onset of the noun, approximately 1,500 ms after verb onset.

**Fig. 5:**
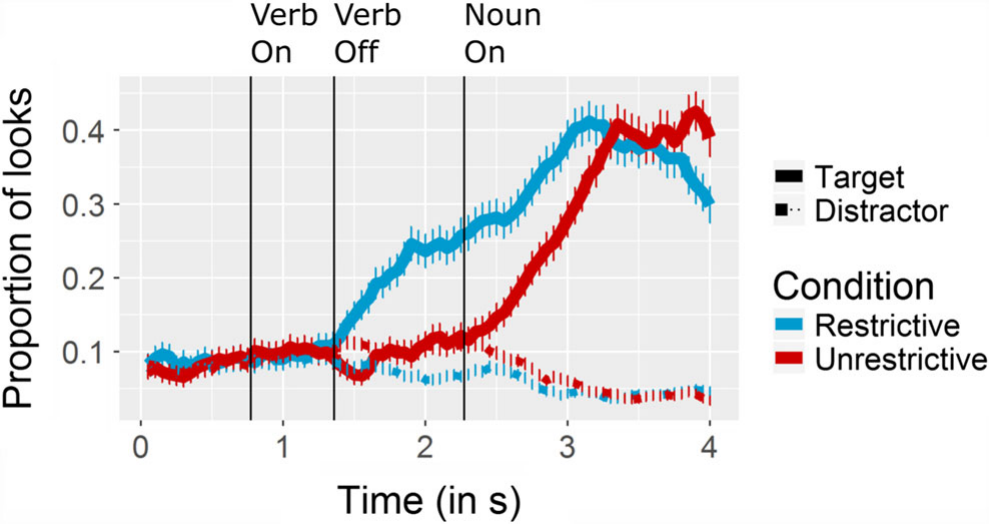
Proportions of looks to the targets and distractors. The collapsed data are averaged across all participants (*N* = 21) and trials. Time 0 represents sentence onset. The vertical lines indicate critical time points averaged across trials. The main statistical analysis was performed on the time window between verb onset (“Verb On”) and noun onset (“Noun On”). Error bars indicate standard errors of the means

The main statistical analysis was hence performed on the critical time window between the verb and noun onsets. We performed a regression analysis using a linear mixed model. As the dependent variable we entered the empirical logits of the proportions of target fixations. We modeled time (as a mean-centered bin), condition (effect-coded), and their interaction as fixed effects, and subject and trial as random effects. The fixation proportions time-locked to sentence onset are illustrated in Fig. 5.

The model revealed that all fixed effects were significant (condition: *Est* = 0.34, *SE* = 0.02, *p* < .001; time: *Est* = 0.02, *SE* = 1.01 × 10^–3^, *p* < .001; Condition × Time: *Est* = 0.02, *SE* = 2.02 × 10^–3^, *p* < .001). This means that fixations to the target increased over time and that the target was fixated more often during the restrictive condition. The significant interaction between condition and time reflected that the increase of target fixations was more pronounced in the restrictive condition.

We performed the same analysis on the mean distractor fixations. The model revealed the same significant effects, but with a reversed sign for the influence of condition (condition: *Est* = –0.16, *SE* = 9.17 × 10^–3^, *p* < .001; time: *Est* = 5.27 × 10^–3^, *SE* = 5.23 × 10^–4^, *p* < .001; Condition × Time: *Est* = –8.41 × 10^–3^, *SE* = 1.06 × 10^–3^, *p* < .001). Visual inspection of the gaze patterns (Fig. 5) suggested that fixations to the distractor objects in the critical time window can be qualitatively described as mirroring the target fixations.

These results are consistent with the hypothesis that participants directed their gazes to the target object more often and significantly earlier in the restrictive than in the unrestrictive condition. The fixations to the distractor objects were influenced in the opposite way, meaning that the fixation proportions to distractors in the restrictive condition decreased over time. This pattern confirms that before noun onset the targets—and not the distractors—attracted more fixations in the restrictive condition only.

### Test for baseline effects

Finally, we analyzed the baseline period (i.e., the interval between time 0 and verb onset) preceding the critical time window, to test whether any confounding interactions were present before the onset of the experimental manipulation. Visual inspection of this baseline period in Fig. 5 suggests that there was no difference in the proportions of looks to the target versus the distractors in this time window. The mean difference between target and distractor fixations across this time window was indeed <.001. We performed a linear regression on the differences in proportions to confirm that the difference did not change over time. The statistical model, which included time as a fixed factor and subject and trial as random factors (*Est* = 6.67 × 10^–3^, *SE* = 0.01, *p* = *.*82), confirmed the absence of differences during the baseline period.

## Discussion

The purpose of the present study was twofold. First, we aimed to conceptually replicate the findings of Altmann and Kamide (1999) in an immersive 3-D VR environment. Second, in doing so, we tested whether it is methodologically feasible to combine VR and eyetracking in the study of online language processing in a multimodal 3-D CAVE environment. Our successful conceptual replication of the original study indicates that the previous findings do generalize to richer situations of stereoscopic 3-D vision. Methodologically, the present study confirms the feasibility of measuring eye movements in a rich 3-D experimental virtual environment, and it may therefore serve as a basis for future implementations that go beyond conceptual replication.

Altmann and Kamide (1999) presented visual stimuli that depicted seminaturalistic scenes. They argued that the predictive relationship they found between an auditorily presented verb and its syntactic arguments was mediated by the real-world context in which the scenes were embedded. The design of the seminaturalistic scenes, however, lacked experimental control of certain critical aspects that are known to influence an observer’s eye movements, such as the direction of the eye gaze of the depicted agent and the animacy of the target object. The fact that we observed a similar effect while controlling for these visual and additional lexical stimulus characteristics confirms the validity of the original effect in these respects. Moreover, we showed that the effects generalize to more naturalistic viewing conditions in which participants observe 3-D objects. The effect of increased target fixations in the restrictive verb condition seems more pronounced in our results than in the original study. This might be related to the lack of filler items in the present study. All spoken sentences referred to the presented scene, and the target object named in the sentence was always visually present.

We also observed a significant effect of experimental condition on the proportions of fixations to the *distractor* objects. Fixation proportions to the distractors in the restrictive condition decreased more strongly than in the unrestrictive condition. Broadly speaking, this pattern mirrored the pattern of looks to the target objects. This is an intuitive result, given the design of the verb lists. The verbs in the restrictive condition are characterized by their semantic features, which make the distractor objects implausible arguments. Altmann and Kamide (1999) found the same tendency but did not characterize the effect statistically. This discrepancy demonstrates that regression models may be more suitable to detecting subtle effects in eyetracking data. The clearer effect in our data can also be attributed to the higher number of trials per condition. Furthermore, the fact that target fixations increased more than distractor fixations decreased can be explained by our definition of ROIs. We coded only for fixations that were directed to the predefined ROIs, and not to other parts of the display. The proportions per time bin therefore do not add up to 1. Fixations to other parts of the display, such as the virtual agent, were not captured in the data, which could account for the remaining proportions.

Previous extensions of Altmann and Kamide’s (1999) paradigm had aimed to test the ecological validity of the original findings by using photographs of visual scenes (e.g., Andersson et al., 2011; Staub et al., 2012). These studies conceptually replicated the original findings, although one study suggested that the original effects may have been restricted to situations in which only a limited number of objects were presented (Sorensen & Bailey, 2007). The present study focused on a different element that is present in naturalistic language processing in a visual context but not in the typical VWP study—namely, the 3-D character of objects that are referred to and the corresponding stereoscopic view that includes natural depth cues. Moreover, unlike in typical screen-based studies using the VWP, our participants were allowed to move their heads, and their visual field was not limited to the fovea. These elements are critical in bridging the gap between traditional experimental paradigms in psycholinguistics and everyday situations of naturalistic language processing in rich multimodal contexts.

In general, the present study adds to the previous evidence suggesting that VR is a promising tool for solving the trade-off between experimental control and ecological validity in psycholinguistic research. A critical assumption for generalizing findings obtained in a VR context to everyday situations is that people behave similarly in similar situations in the virtual world and the real world. Initial studies into language processing indicated that this assumption was met. Similar linguistic-priming effects occurred when participants interacted with either a human-like virtual agent or a real person (Heyselaar et al., 2017), and participants accommodated their speech rate and pitch to the speech rate and pitch of their virtual interlocutors (Gijssels et al., 2016; Staum Casasanto et al., 2010). Other recent studies have also suggested that participants behave similarly in producing and comprehending language in VR versus traditional experimental paradigms, in terms of both behavioral and neurophysiological measures (Peeters & Dijkstra, 2017; Tromp et al., in press). The present study is in line with this overall tendency, in showing that participants predicted upcoming words in virtual contexts qualitatively similarly to predictions of upcoming words in traditional, nonimmersive experimental paradigms. Future studies performed in CAVE environments may combine eyetracking with recording of electrophysiological data in order to further investigate the neurocognitive underpinnings of prediction in rich, multimodal contexts.

At a methodological level, we showed the feasibility of combining eyetracking and VR in a CAVE environment. A technical issue that may be improved in future implementations is the test for tracking accuracy of the in-built eyetracking device. For the present setup it was not possible to assess tracking accuracy quantitatively, but only via a warning message from the tracking software during the initial calibration step. When tracking accuracy was found to be too low, those participants were excluded from the study. Additional assessments of tracking accuracy and calibration performance were performed offline using playback software to visually inspect the gaze patterns. Participant exclusion should ideally be performed by assessing tracking accuracy quantitatively over the course of the whole experiment.

In sum, the present study showed verb-mediated predictive language processing in a rich, multimodal 3-D environment, thereby confirming the ecological validity of previous findings in nonimmersive 2-D environments. We conclude that eyetracking measures can reliably be used to study theoretically interesting phenomena in automated virtual environments that allow for richer and ecologically more valid forms of stimulus presentation than do traditional, screen-based experimental paradigms.

### Electronic supplementary material

Below is the link to the electronic supplementary material.

ESM 1 (DOCX 351 kb)

## Appendix 1. Linguistic parameters of the verbs

Table 2

**Table 2:** Lexical characteristics of the verb materials in the restrictive and unrestrictive conditions

Measure	Restrictive	Unrestrictive	*p* Value
Length (*# letters)*	6.00 (1.48)	6.50 (0.74)	.43
Length (# *syllables*)	2.00 (0.00)	2.00 (0.00)	.25
Length (# *phonemes*)	5.00 (1.48)	5.00 (1.48)	.47
Frequency (SUBTLEX-NL) (log)*	2.93 (0.63)	3.05 (0.68)	.46
Frequency (SUBTLEX-NL2) (log)*	3.40 (0.63)	3.53 (0.68)	.42
Similarity to other words	1.48 (0.52)	1.45 (0.52)	.40
Age of acquisition	6.21 (1.39)	6.72 (1.74)	.32
Word prevalence	2.75 (0.21)	2.80 (0.38)	.95
Reaction time	526.50 (31.88)	520.00 (22.98)	.81
Accuracy	.98 (.03)	.98 (.03)	.52
Coltheart *N*	7.50 (8.15)	7.00 (7.41)	.52

Normally distributed parameters are marked with an asterisk (*). Listed are the mean values with *SD*s, for normally distributed parameters, or median values with median absolute deviations, otherwise.

## Appendix 2. Stimulus materials

Table 3

**Table 3:** List of verb pairs and the four object names for each scene, all in Dutch

Scene No.	Restrictive Verb	Unrestrictive Verb	Target Object	Distractor 1	Distractor 2	Distractor 3
1	bakken	bezorgen	frietjes	kruk	microfoon	tennisbal
2	bespelen	reinigen	piano	tandenborstel	kachel	spiegel
3	besturen	wassen	auto	broek	kom	schep
4	breken	vergeten	wijnfles	rekenmachine	trui	muts
5	dekken	poetsen	tafel	wereldbol	schoenen	map
6	drinken	bereiden	koffie	biefstuk	tomaat	mais
7	duwen	bezien	kinderwagen	wafel	poster	open haard
8	eten	dragen	watermeloen	gieter	stoel	halter
9	horen	testen	telefoon	lippenstift	pan	basketbal
10	installeren	winnen	computer	elpee	bloem	voetbal
11	kappen	filmen	boom	sporttas	paraplu	koffer
12	knopen	grijpen	stropdas	hamburger	aansteker	kam
13	koken	bekijken	pompoen	ballon	viool	wasbak
14	kraken	nuttigen	walnoot	donut	tompouce	muffin
15	lezen	kopen	krant	druiven	oven	gitaar
16	likken	kiezen	lolly	zaag	pion	veer
17	openen	beschrijven	deur	mand	thee	hoed
18	persen	vangen	sinaasappel	magneet	pijp	bril
19	plukken	nemen	tomaatje	ijsje	bord	horloge
20	repareren	stelen	fietspomp	sigaar	dennenappel	banaan
21	roken	pakken	sigaretten	beker	fiets	tas
22	roosteren	ontvangen	boterham	vlag	jas	schaar
23	ruiken	gooien	parfum	mes	skateboard	cadeau
24	schillen	wegen	appel	emmer	doos	sleutel
25	slikken	checken	pillen	koptelefoon	laptop	bel
26	sluiten	begeren	boek	kopje	papier	ei
27	smelten	zoeken	chocolade	ontstopper	mobiel	gum
28	snijden	proeven	taart	soep	wijn	cocktail
29	stoppen	lenen	radio	zonnebril	kaars	pen
30	tellen	verstoppen	ballen	hamer	lamp	paprika
31	versturen	verplaatsen	brief	kleerhanger	liniaal	TV
32	volgen	tekenen	wegwijzer	pizza	kan	bezem

Depending on the experimental condition, either the restrictive or the unrestrictive verb was used in the spoken sentence.

Table 4

**Table 4:** Stimulus materials translated into English

Scene No.	Restrictive Verb	Unrestrictive Verb	Target Object	Distractor 1	Distractor 2	Distractor 3
1	bake	deliver	fries	stool	microphone	tennis ball
2	play	clean	piano	toothbrush	stove	mirror
3	drive	wash	car	pants	bowl	shovel
4	break	forget	wine bottle	calculator	jumper	hat
5	set	clean	table	globe	shoes	folder
6	drink	prepare	coffee	steak	tomato	corn
7	push	look at	buggy	waffle	poster	fireplace
8	eat	carry	watermelon	watering can	chair	barbell
9	hear	test	telephone	lipstick	pan	basketball
10	install	win	computer	LP	flower	football
11	cut	film	tree	sports bag	umbrella	suitcase
12	knot	grab	tie	hamburger	lighter	comb
13	cook	look at	pumpkin	balloon	violin	sink
14	crack	eat	walnut	donuts	cake	muffin
15	read	buy	newspaper	grapes	oven	guitar
16	lick	choose	lolly	saw	pawn	feather
17	open	describe	door	basket	tea	hat
18	squeeze	catch	orange	magnet	pipe	glasses
19	pick	take	tomato	ice cream	plate	watch
20	fix	steal	bike pump	cigar	pine cone	banana
21	smoke	take	cigarettes	cup	bike	bag
22	roast	get	slice of bread	flag	jacket	scissors
23	smoke	throw	perfume	knife	skateboard	gift
24	peel	weigh	apple	bucket	box	key
25	swallow	check	pills	head phone	laptop	bell
26	close	want	book	cup	paper	egg
27	melt	search	chocolate	plunger	mobile phone	eraser
28	cut	taste	cake	soup	wine	cocktail
29	stop	borrow	radio	sunglasses	candle	pencil
30	count	hide	balls	hammer	lamp	paprika
31	send	move	letter	hanger	ruler	television
32	follow	draw	sign	pizza	can	broom
