# Supplementary material for: Language-driven anticipatory eye movements in virtual reality
Source: Behav Res Methods. 2017 Aug 8;50(3):1102–15. doi: 10.3758/s13428-017-0929-z (PMC5990548; doi:10.3758/s13428-017-0929-z)
Supplement: Supplementary file 1 — (DOCX 351 kb) [file 13428_2017_929_MOESM1_ESM.docx]

**Supplementary materials**

**
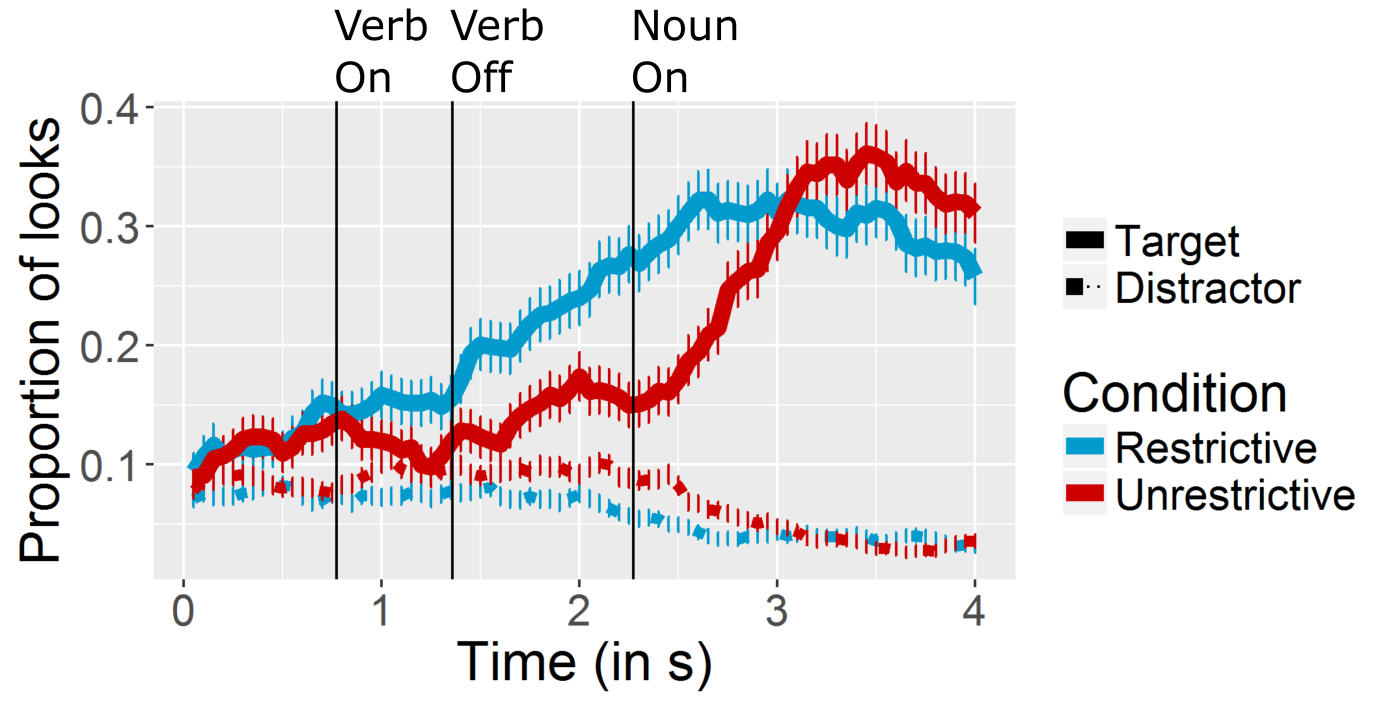
**

**Figure S1.** Proportion of looks to targets and distractors in Block 2 (excluded from the main analysis). The collapsed data are averaged across all subjects (*N* = 21) and trials. Time 0 represents sentence onset. Vertical lines indicate critical time points averaged across trials. Error bars indicate standard errors of the mean.


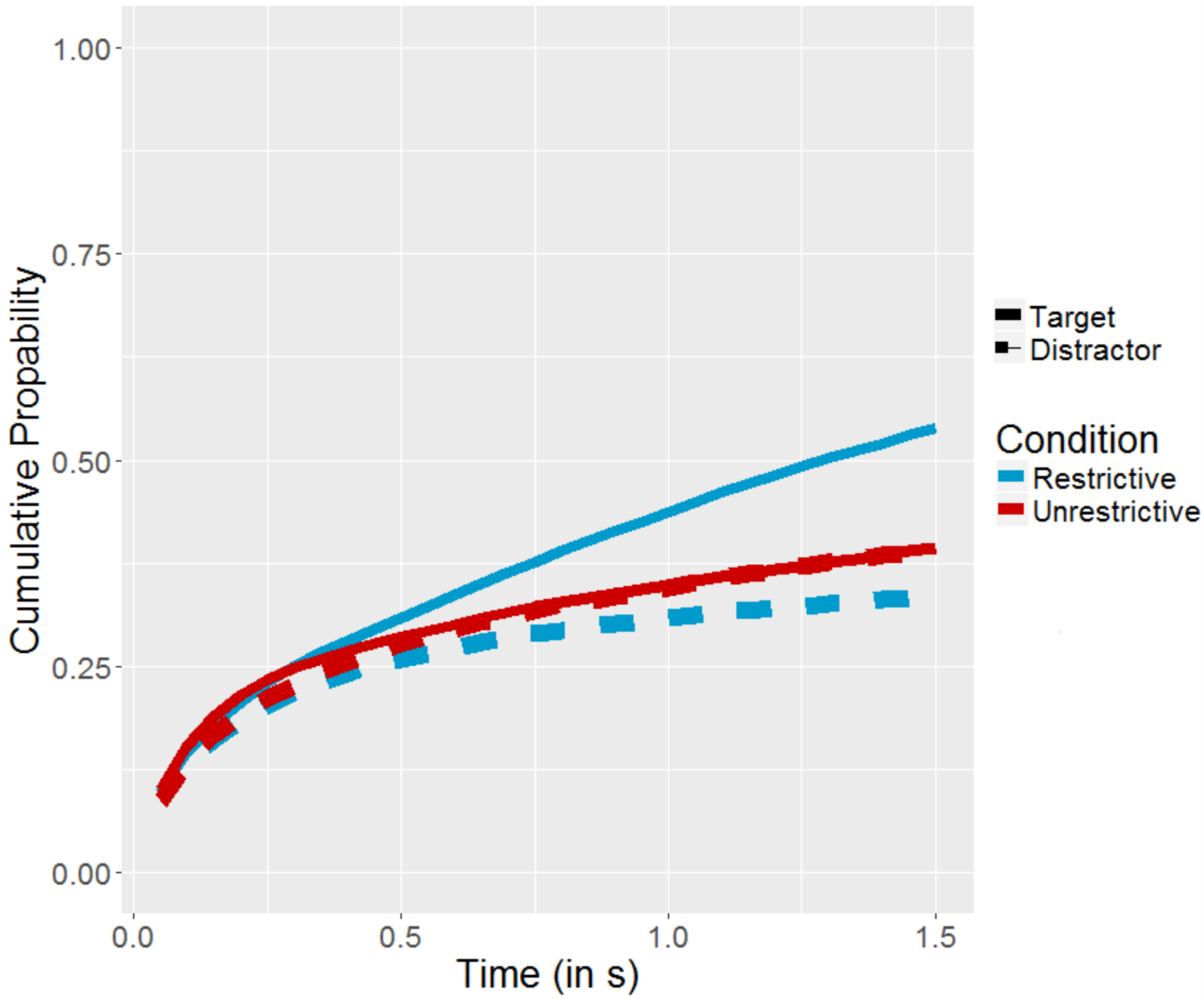


**Figure S2.** Cumulative probability of looks to target and distractors during the critical time window between verb onset and noun onset. Time 0 represents verb onset. Data points for all subjects (*N* = 21) and trials were collapsed and weighted equally. Figure for comparison with Altmann & Kamide (1999)'s Figure 2.
